# Supplementary material for: Precursor region with full phonon softening above the charge-density-wave phase transition in 2H-TaSe2
Source: Nat Commun. 2023 Nov 10;14:7282. doi: 10.1038/s41467-023-43094-5 (PMC10638379; doi:10.1038/s41467-023-43094-5)
Supplement: Supplementary file 1 — Supplementary Information [file 41467_2023_43094_MOESM1_ESM.pdf]

Supplementary Information to:

Precursor region with full phonon softening above the charge-density-wave phase transition in 2*H*-$TaSe_2$

Xingchen Shen[1,2], Rolf Heid[1], Roland Hott[1], Amir-Abbas Haghighirad[1], Björn Salzmann[3], Marli dos Reis Cantarino[3,4], Claude Monney[3], Ayman H. Said[5], Mehdi Frachet[1], Bridget Murphy[6,7], Kai Rossnagel[6,7,8], Stephan Rosenkranz[9], Frank Weber[1,✉]

[1] Institute for Quantum Materials and Technologies, Karlsruhe Institute of Technology, 76021 Karlsruhe, Germany
[2] College of Physics, Chongqing University, Chongqing 401331, P. R. China
[3] Département de Physique and Fribourg Center for Nanomaterials, Université de Fribourg, 1700 Fribourg, Switzerland
[4] Instituto de Física, Universidade de São Paulo, São Paulo-São Paulo 05508-090, Brazil
[5] Advanced Photon Source, Argonne National Laboratory, Lemont, Illinois 60439, USA
[6] Institute of Experimental and Applied Physic and KiNSIS, Kiel University, 24098 Kiel, Germany
[7] Ruprecht Haensel Laboratory, Kiel University, 24098 Kiel, Germany
[8] Ruprecht Haensel Laboratory, Deutsches Elektronen-Synchrotron DESY, 22607 Hamburg, Germany Germany
[9] Materials Science Division, Argonne National Laboratory, Lemont, Illinois 60439, USA

✉ *frank.weber@kit.edu*

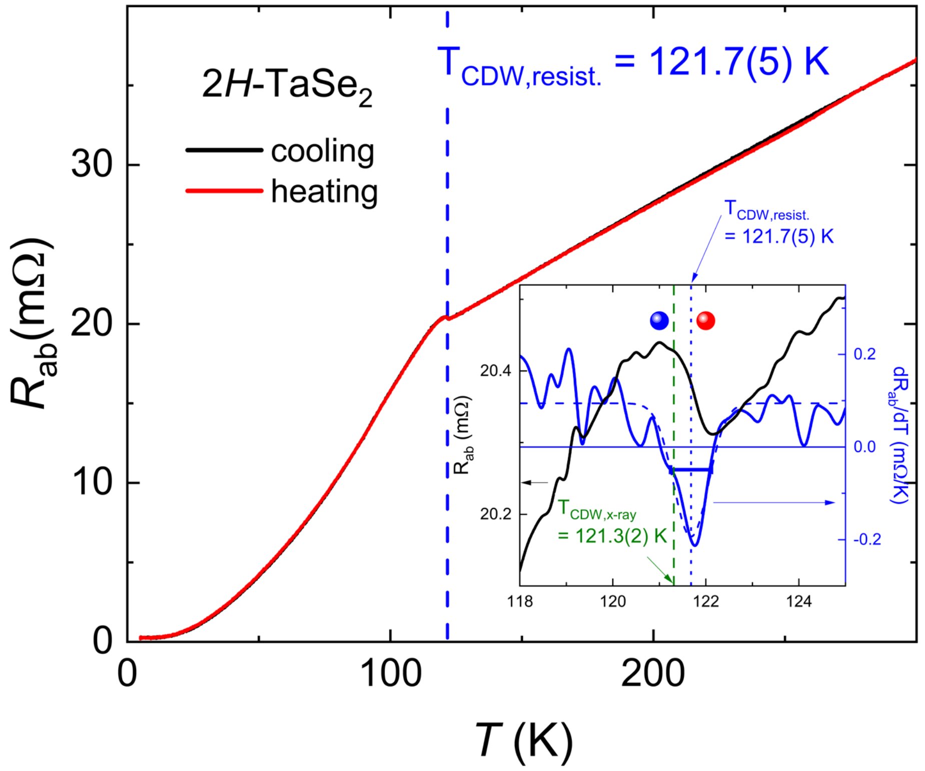

Fig. S1. (a) High resolution In-plane electrical resistance of 2*H*-$TaSe_2$ as a function of temperature. The red and black curves correspond to heating and cooling conditions, respectively. No significant thermal hysteresis is observed from room to base temperature. The residual resistivity ratio, *R*(300K)/*R*(T->0), amounts to 143. (inset) Zoom around T_{CDW}, where a sharp resistance upturn upon cooling signals the onset of the charge-density-wave state (black solid line, left-hand scale). $T_{CDW,resist.}$ = 121.7(5) (vertical dotted line) was determined from a fit (dashed blue line, right-hand scale) to dR_{ab}/dT (blue solid line, right-hand scale). The full-width-at-half-maximum of the approximated negative peak in dR_{ab}/dT (blue thick horizontal bar) was taken as the uncertainty of ±0.5 K for $T_{CDW,resist.}$. The dashed vertical line denotes $T_{CDW,x\text{-}ray}$ deduced from our x-ray measurements [see inset in Fig. 5(b)]. The blue and red spheres denote the temperatures T = 121 K and 122 K, respectively, at which we performed x-ray measurements clearly below and above the CDW transition temperature [see same symbol code in Fig. 5(b)-(d)].

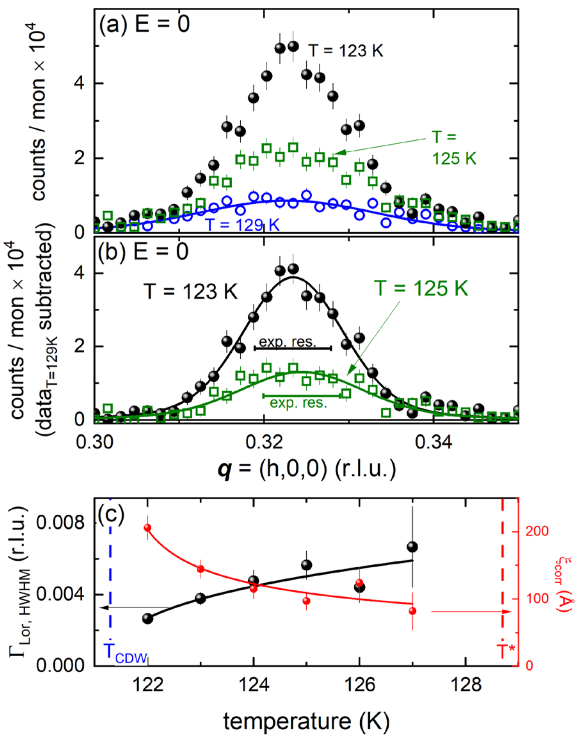

Fig. S2. (a) High resolution momentum scans at zero energy transfer for T = 123 K (black spheres) and 125 K (green open squares) and T =129 K (≈ T*, blue circles). The solid blue line denotes a fit to the data at T = 129 K and was subtracted from the data taken at lower temperatures as phonon dominated background (see text). (b) Background subtracted high resolution momentum scans at zero energy transfer for T = 123 K (black spheres) and 125 K (green open squares). Solid lines are approximated Voigt functions where the Gaussian widths were fixed to the experimental resolution (FWHM indicated by the horizontal bars). (c) Temperature-dependent line width of the Lorentzian part of the Voigt function, $\Gamma_{Lor,HWHM}$ (black spheres, left-hand scale). The corresponding correlation length $\xi_{corr} = a/(2\pi \times \Gamma_{Lor,HWHM})$ is shown in red (right-hand scale). Solid lines are guides to the eye. The vertical blue and red dashed lines denote T_{CDW} = 121.3 K and T* = 128.7 K, respectively.

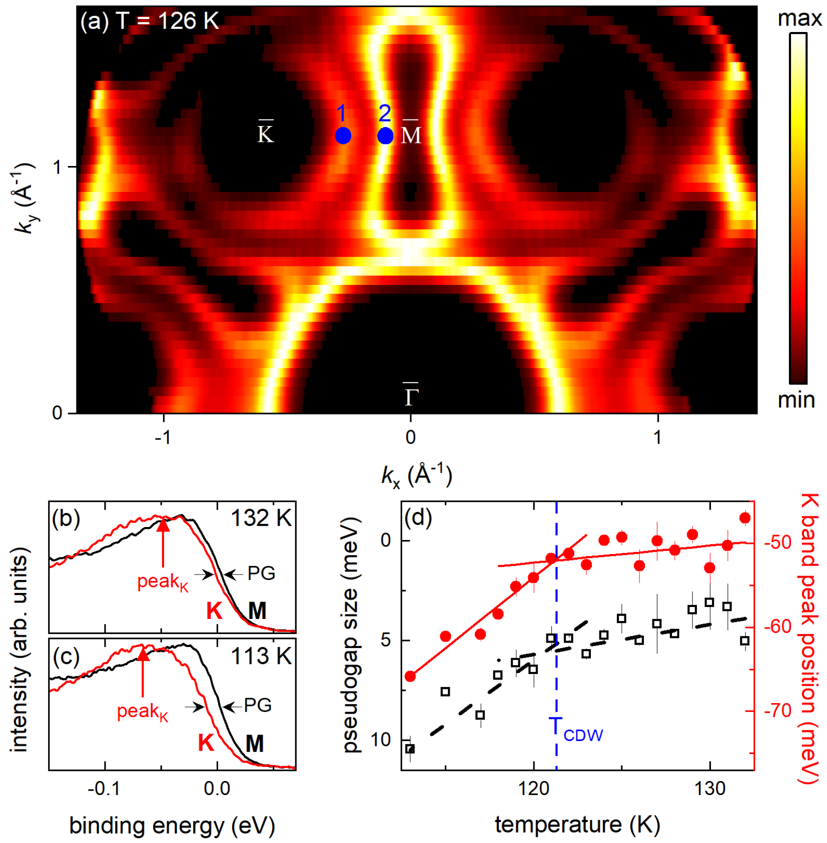

FIG. S3. (a) Fermi surface map measured at T = 126 K. White characters denote high-symmetry points of the Brillouin zone. Blue points 1 and 2 indicate the momentum position of the EDCs shown in red and black, respectively, in panels (b) and (c). (b),(c) EDCs at (b) T = 132 K and (c) 113 K obtained at the FS pockets around the $\bar{K}$ point [red solid line, blue point #1 in (a)] and the $\bar{M}$ point [black solid line, blue point #2 in (a)]. The horizontal and vertical arrows indicate the deduced sizes of the pseudo-gap and $\bar{K}$-band peak position, respectively. (d) Temperature dependence of the pseudo-gap (open squares) and $\bar{K}$-band peak position (dots). Lines are linear fits to the data for $T < T_{CDW}$ and $T > T_{CDW}$. The vertical blue dashed line denotes T_{CDW} = 121.3 K deduced from elastic x-ray scattering.

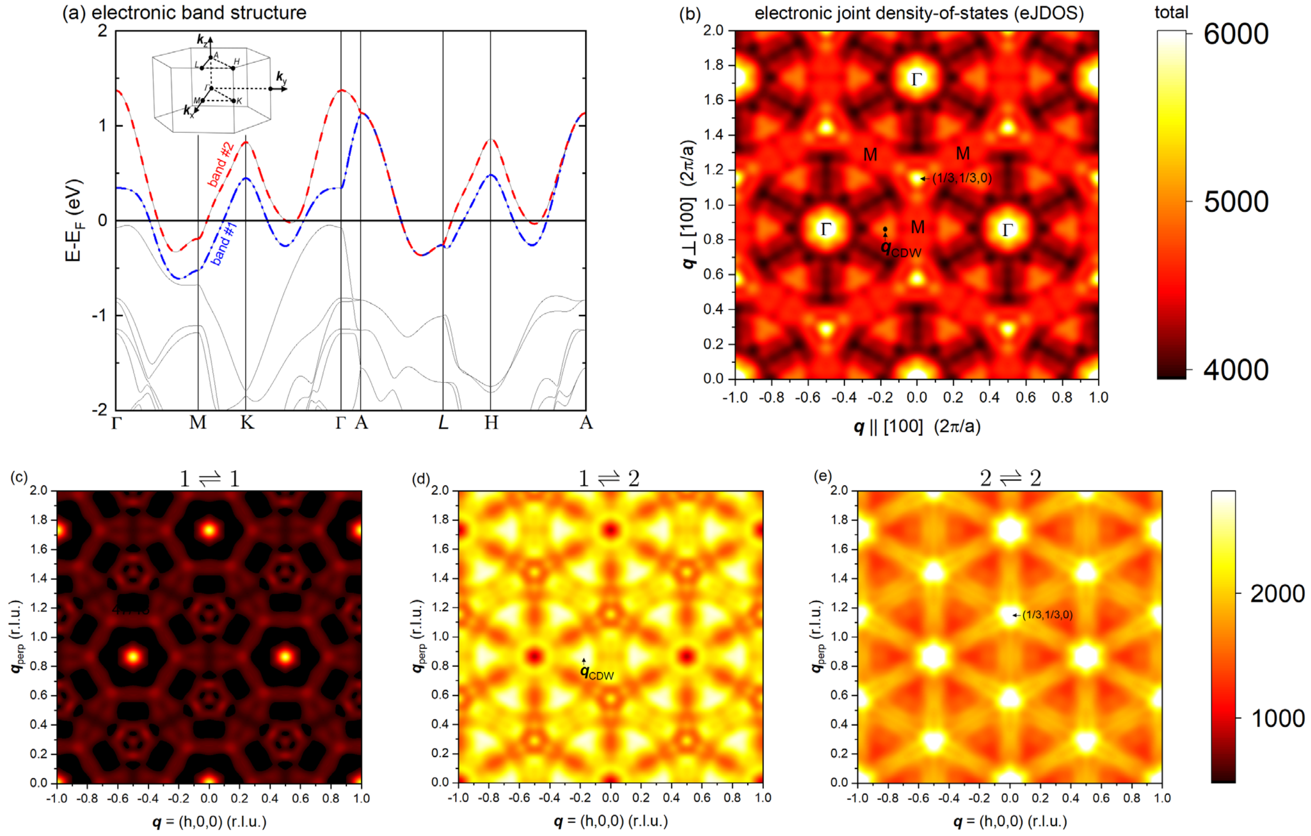

FIG. S4. (a) Calculated electronic band structure of 2*H*-$TaSe_2$ along several high-symmetry directions. Two-fold degenerate bands making up the Fermi surface are labelled as described in the text. The inset shows a sketch of the Brillouin zone of 2*H*-$TaSe_2$. (b) Calculated total electronic joint density-of-states (eJDOS) in the $\boldsymbol{q} = (h, k, 0)$ plane. Letters denote high symmetry points of the Brillouin zone. One position of $\boldsymbol{q}_{CDW} = (0.324,0,0)$ is indicated. $\boldsymbol{q} = (1/3,1/3,0)$ indicates the strongest Fermi surface nesting feature. (c)-(e) eJDOS for specific band transitions as indicated in the title of the panels. The range in $\boldsymbol{q}$ is always the same as in panel (a).

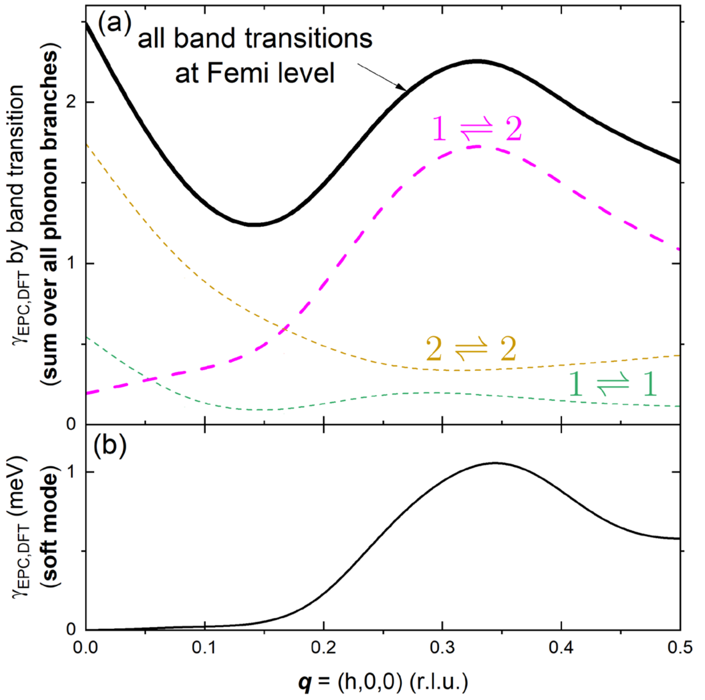

FIG. S5. (a) Calculated $\gamma_{EPC,DFT}$ (FWHM) along $\Gamma - M$ including contributions from transitions between all bands at the Fermi level (solid line). Dashed lines denote $\gamma_{EPC,DFT}$ related to specific band transitions given in the panel. (b) $\gamma_{EPC,DFT}(\boldsymbol{q})$ along $\Gamma - M$ for the LA soft phonon mode.

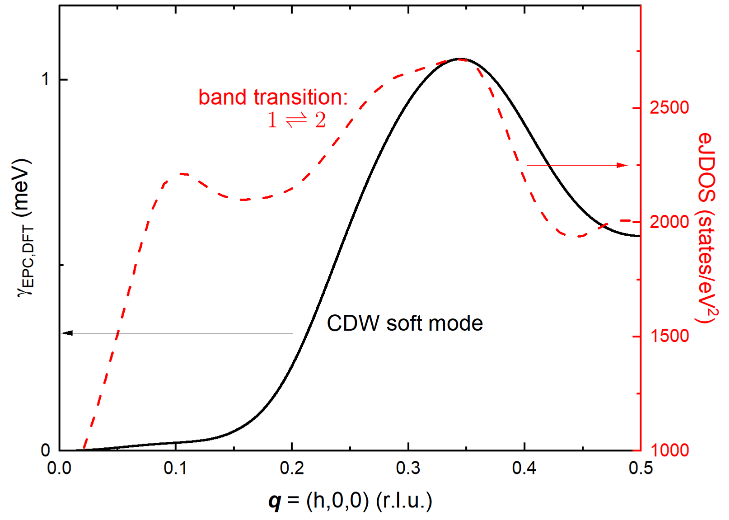

FIG. S6. $\gamma_{EPC,DFT}(\boldsymbol{q})$ for the LA soft phonon mode (solid line, left-hand scale) and the eJDOS for the band transition $1 \leftrightarrow 2$ (dashed line, right-hand scale) along the $\Gamma - M$ line.

Supplemental Note 1 : Resistivity

Electrical resistance measurements were done on a sample from the same growth batch as that investigated by IXS in a standard four-point geometry, using a combination of Keithley 6221 current source and Keithley 2182A nanovoltmeter in delta mode. Electrical contacts were made using DuPont 4929N silver paint. Measurements were performed using a helium bath cryostat, with the sample under low pressure (mBar) helium atmosphere, and a slow ramping rate of 1K per minute. Temperature was measured by a calibrated cernox sensor attached close to the sample on the probe insert. The CDW transition temperature was determined as the minimum of the derivative, dR/dT, to $T_{CDW,resist.}$ = (121.7 ± 0.5) K in close proximity to that deduced from our x-ray measurements, i.e., $T_{CDW,x-ray}$ = (121.3 ± 0.2) K. The residual resistivity ratio, *R*(300K)/*R*(T->0) = 143, is larger than values reported in other publications[1,2] and indicates, from a transport perspective, a high quality of our samples.

Supplemental Note 2 : Inelastic x-ray scattering

The IXS experiments were carried out at the 30-ID beamline, HERIX spectrometer[3], at the Advanced Photon Source, Argonne National Laboratory, with a focused beam size of 15 µm × 32 µm. The incident energy was 23.724 keV [4] and the horizontally scattered beam was analyzed by a set of spherically curved silicon analyzers (Reflection 12 12 12) [5]. The full width at half maximum (FWHM) of the energy and wave vector space resolution was about 1.5 meV and 0.09 $Å^{-1}$, respectively, where the former is experimentally determined by scanning the elastic line of a piece of Plexiglas and the latter is calculated from the experiment geometry and incident energy. Phonon excitations measured in constant-momentum scans were approximated by damped harmonic oscillator (DHO) functions[6] convoluted with a pseudo-voigt resolution function (FWHM = 1.5 meV). The resolution function was further used to approximate resolution limited elastic scattering at zero energy transfer. Measurements were done at scattering wave vectors $\mathbf{Q} = \boldsymbol{\tau} - \mathbf{q}$, where $\boldsymbol{\tau}$ is a reciprocal lattice vector and $\boldsymbol{q}$ is the reduced wave vector in the 1st Brillouin zone. Wave vectors are expressed in reciprocal lattice units (r.l.u.) $(2\pi/a, 2\pi/b, 2\pi/c)$ with the lattice constants $a = b = 3.44$ Å and $c = 12.7$ Å of the high-temperature hexagonal unit cell (#194). All measurements were done in the Brillouin zone adjacent to $\boldsymbol{\tau} = (3,0,1)$. Results are presented in reduced wave vectors $\boldsymbol{q} = \boldsymbol{\tau} - \boldsymbol{Q}$. We used a high-quality single crystal sample grown at Kiel University weighing about 5 mg (2 x 2 x 0.02 mm^3). The sample was mounted on a thin sheet of Be attached to the cold finger of a closed-cycle refrigerator (CCR from Advanced Research Systems, ARS) via a Cu sample holder. The samples were about 20 µm thin and fragile. Thus, the Be sheet stabilizes the samples and provides a good thermal contact. The Be sheet itself (thickness: 125 µm) is practically transparent to hard x-rays with 23.724 keV because the thickness is more than two orders of magnitude smaller than the absorption length. Measurements reported here were done at various temperatures $60\,\mathrm{K} \leq T \leq 300\,\mathrm{K}$ and the temperature was measured via a calibrated Si diode sensor attached to the cold finger of the CCR.

Measured energy spectra at constant momentum transfer were approximated using a pseudo-Voigt function for the resolution limited elastic peak and a damped harmonic oscillator (DHO) function [6] for the phonon peaks. The DHO function was convoluted with the fit of the experimental resolution function. The DHO function is

$$S(Q,\omega) = \frac{[n(\omega)+1]Z(Q)4\omega\Gamma/\pi}{\left[\omega^2 - \tilde{\omega}_q^2\right]^2 + 4\omega^2\Gamma^2} \qquad (1)$$

where $\boldsymbol{Q}$ and ω are the wavevector and energy transfer, respectively, n(ω) is the Bose function, Γ is the imaginary part of the phonon self-energy, $\tilde{\omega}_q$is the phonon energy renormalized by the real part of the phonon self-energy and $Z(\boldsymbol{Q})$ is the phonon structure factor. This function covers the energy loss and energy gain scattering by a single line shape. The intensity ratio of the phonon peaks at $E = \pm\omega_q$ is fixed by the principle of detailed balance. Thus, the fit function for the same mode at energy loss and energy gain, *e.g.*, the soft phonon mode [see solid blue lines in Figs. 2 and 4], features only three free parameters: $\tilde{\omega}_q$, Γ and the phonon intensity Z. The energy ω_q of the damped phonons is obtained from the fit parameters of the DHO function by $\omega_q = \sqrt{\tilde{\omega}_q^2 - \Gamma^2}$ [7].

Energy and momentum resolution are coupled in measurements of dispersing phonon modes and there will be an additional contribution to the peak's linewidth. The contribution is larger for stronger dispersing modes, i.e., it

increases for the measurements of the soft phonon mode near the phase transition temperature where the dispersion is steepest [see Fig. 3(c)]. Thus, the large discrepancy between the deduced and calculated phonon linewidths originates – at least partially – from this effect [see Fig. 6(b)]. A quantitative calculation of this effect would require the convolution of the (unknown) three-dimensional dispersion of the soft mode with the independent momentum and energy resolution functions including full structure factor calculations. This is outside the scope of this work.

On the other hand, the phonon intensity scales with the inverse of the phonon energy, e.g., a phonon at E = 0.4 meV has five times more intensity as a mode at 2 meV (assuming a constant structure factor). Hence, the peak position of the soft mode measured at $\boldsymbol{q}_{CDW}$ will be dominated by the lowest energy mode in the dispersion. These considerations also explain that a resolution-induced error in the determination of the phonon energy will result in values larger than the real energy of the phonon at the minimum of the dispersion at $\boldsymbol{q}_{CDW}$, i.e., our analysis provides an upper limit for the energy of the soft phonon mode. Thus, this effect cannot explain the softening of the LA phonon at $\boldsymbol{q}_{CDW}$ already well above T_{CDW}.

We note that IXS spectra generally contain a small, resolution-limited peak at zero energy transfer due to crystal imperfections. In our sample of 2*H*-$TaSe_2$ this defect scattering peak is tiny with amplitudes < 1 count/mon×10^5 observed at temperatures far above T_{CDW} [Figs. 2(a)-(c)], confirming its high quality. Below T < 150 K , the zero energy transfer intensity at $\boldsymbol{q}_{CDW}$ starts to slowly rise due to the onset of the static CDW superlattice peak. For T = 130 K, the data exhibit a resolution-limited peak at E = 0 [dash-dotted (black) line in Fig. 2(e)] with an amplitude that is ten times the value of the elastic line due to crystal imperfection observed at temperatures T > 150 K. Yet, this intensity is still small compared to that of the soft phonon mode [solid (blue) line in Fig. 2(e)]. The static superlattice peak becomes similarly strong as the overdamped phonon in scattering intensity only below T* = 128.7 K.

IXS is not able to detect phonon intensities when there is strong elastic scattering, *e.g.*, at a reciprocal lattice point or at $\boldsymbol{q}_{CDW}$ (for $T < T_{CDW}$). Therefore, the CDW soft phonon mode in 2*H*-$TaSe_2$ cannot be investigated anymore for $T \leq T_{CDW}$ due to the rise of the superlattice peak at $\boldsymbol{q}_{CDW}$. However, neighboring wave vectors are not affected by the superlattice peak. The phonon softening is detectable at $\boldsymbol{q}$ = (0.28,0,0) where the LA phonon energy softens by about 2 meV between T = 250 K and 130 K [Fig. 3(a)]. Cooling below T_{CDW}, we observe a hardening and narrowing of the phonon mode in the CDW phase. The temperature dependence of phonon energy levels off around $T_{C\text{-}IC} \approx 90$ K in reasonable agreement with results from Raman spectroscopy of the CDW soft phonon for $T < T_{CDW}$.[8]

In the following and Figure 6, we explain in detail the analysis of the momentum scans from which we deduce the correlation length of the CDW domains for $T_{CDW} < T < T^*$ [see Fig. 5(e)]. Momentum scans at zero energy transfer at $\boldsymbol{q}$ = (0.3 – 0.37,0,0) were performed with the best momentum resolution possible on the HERIX spectrometer, Δq = 0.018 Å^{-1}. Here, we decreased the effective size of the backscattering analyzers by closing a circular slit to a diameter of 18 mm (FWHM) compared to 95 mm opening in regular inelastic scans. From a fit we can determine the line width $\Gamma_{exp,FWHM}(T)$ [see Fig. 5(d)]. However, we cannot simply take the analysis of $\Gamma_{exp,FWHM}(T)$ at face value to analyze the CDW correlation length because our energy scans demonstrate the significant two-component nature of the scattering at zero energy transfer for $T > T_{CDW}$ [see Fig. 4(a)-(c)]. On the other hand, the energy scans [see Fig. 4] also show that the scattering from the soft phonon mode (1) dominates for T ≥ T* and (2) is essentially constant for T ≤ T*. We conclude that scattering observed in momentum scans at zero energy transfer is mostly due to the soft phonon mode for T ≥ T*. Therefore, data taken at T = 129 K [blue circles in Fig. S2(a)] represent an estimate of the phononic background in our momentum scans and a fit [solid blue line in Fig. S2(a)] was subtracted from data taken at lower temperatures. The resulting scans indicate the evolution of the static CDW superlattice peak only [Fig. S2(b)]. Thereby, we can investigate the correlation length ξ_{corr} of the static CDW superlattice peak at T ≤ T* by approximating the resulting phonon-corrected momentum scans with a Voigt function. We fixed the Gaussian width of the Voigt function to the experimental resolution [see Fig. 5(d)]. The temperature

dependences of the Lorentzian linewidth $\Gamma_{Lor,HWHM}(T)$ and the corresponding $\xi_{corr} = a/(2\pi \times \Gamma_{Lor,HWHM})$ are shown as black and red spheres in Figure 5(e), respectively. ξ_{corr} increases below T* to about 200 Å just above T_{CDW}. Thus, the precursor region in 2*H*-$TaSe_2$ is characterized by medium-range-sized CDW domains, which only form a long-range CDW ordered state at $T \leq T_{CDW}$.

Supplemental Note 3: Angle-resolved photoemission spectroscopy

Angle-resolved photoemission spectroscopy (ARPES) is one of the most powerful experimental techniques to study the electronic band structure of solids. In CDW materials, ARPES has been indispensable to investigate the gap in the electronic excitation spectrum upon entering the ordered phase. Previous ARPES measurements in 2*H*-$TaSe_2$ reported some anomalous behaviour: The band gap observed in energy distribution curves (EDCs) opens only below the onset of commensurate CDW order at $T_{C\text{-}IC} \approx 90$ K.[9] Subsequent studies[10,11] corroborated this observation but reported the opening of a pseudo-gap already on cooling below T_{CDW}. The pseudo-gap opens in the bands of the K barrel and its size can be determined from the shift of the leading edge of the EDC. More details are given below in the analysis of our own data set.

In our experiment, we wanted to check the temperature evolution of the FS in 2*H*-$TaSe_2$ in a narrow temperature range focusing on T_{CDW} and T* in our own samples. We do not discuss more details of the Fermi surface properties and general electronic band structure which were reported on in previous publications[9-12]. ARPES measurements at temperatures 132 K ≥ T ≥ 113 K were performed at the Bloch endstation of the R1 synchrotron at the MAX IV laboratory in Lund, Sweden, using linearly polarized light with 80 eV photon energy with a total resolution of < 10 meV in energy and < 0.2° in angle. The spot size was about 15 µm × 10 µm. The sample was cleaved using tape at 10^{-8} mbar and measurements were performed at < 10^{-10} mbar. Photoelectrons were recorded using a ScientaOmicron DA30-L hemispherical analyser. Sample orientation and cooling were achieved with a six axis "Carving" manipulator from SPECS GmbH cooled by a closed-loop liquid helium cryostat. Sample temperature was determined from previous calibration measurements. After each change in temperature setpoint, the sample temperature was allowed to stabilise for 20 minutes before the next measurement was performed. The sample used for ARPES measurements was a different single crystal from the same growth batch as that used for IXS measurements.

In agreement with previous studies[9,10], we observe hole-like circular FS sections centered on the $\bar{\Gamma}$ and $\bar{\mathrm{K}}$ points and electron-like "dogbones" around the $\bar{\mathrm{M}}$ point [Fig. S3(a)]. Here, $\bar{\Gamma}$, $\bar{\mathrm{K}}$, and $\bar{\mathrm{M}}$ denote the positions of high-symmetry points projected to the basal plane, i.e. $k_z = 0$.

EDCs were taken from ARPES spectra centred around *M* along the $\bar{\mathrm{K}}$-$\bar{\mathrm{M}}$ direction [see blue dots #1 and #2 in Fig. S3(a)] as determined from Fermi surface maps taken at every temperature step. Each EDC is integrated over a 1° range. Each EDC was approximated with a single Gaussian peak modified by a Fermi-Dirac distribution with the Fermi level (E_F) kept constant across all temperatures in order to determine the $\bar{\mathrm{K}}$ barrel peak positionand and the position of the leading edge for $\bar{\mathrm{K}}$ barrel and $\bar{\mathrm{M}}$ dogbone [***k*** corresponding to blue dot #1 in Fig. S3(a)]. We observe good agreement with the data down to at least -0.1 eV binding energy for all spectra. The pseudo-gap size is defined as the difference of the energies at half-height in EDCs taken on the $\bar{\mathrm{K}}$ barrel [blue dot #1 in Fig. S3(a)] and the $\bar{\mathrm{M}}$ dogbone [blue dot #2 in Fig. S3(a)]. Examples for T = 132 K and 113 K [Figs. 7(b) and (c)] are normalised for ease of comparison and the horizontal arrows denote the position at which the pseudo-gap size was defined. The obtained temperature dependences are summarized in Fig. S3(d). The shown values of the $\bar{\mathrm{K}}$ barrel peak position (red dots, right-hand scale) and pseudogap size (black open squares, left hand scale) represent the average of the values obtained for the two pairs of $\bar{\mathrm{K}}$ and $\bar{\mathrm{M}}$ bands at positive and negative momentum relative to the $\bar{\mathrm{M}}$ point visible in Fig. S3(a). The individual values show qualitatively the same behaviour across the studied temperature range as their average shown here. For both the $\bar{\mathrm{K}}$ band peak position and the pseudo-gap size, we observe different slopes in their temperature dependence at low and high temperatures in our data set. Linear fits in each region [solid/dashed lines in Fig. S3(d)] cross close to T_{CDW} = 121.3 K, i.e., the transition temperature deduced from x-ray momentum scans at zero energy transfer [see Fig. 4]. The observed

kinks in the temperature dependence of the pseudogap and $\overline{\mathrm{K}}$-band peak at T_{CDW} agree also with previous results[10]. Finally, our analysis of the ARPES measurements reveals no particular electronic changes at T*.

Supplemental Note 4: *Ab-initio* calculations

Calculations using density-functional-perturbation-theory (DFPT) were performed in the framework of the mixed basis pseudopotential method [42]. Norm-conserving pseudopotentials for Ta and Se were constructed including 5s and 5p semicore states in the valence space in the case of Ta. The deep potentials can be efficiently treated in the mixed-basis scheme, which combines local functions together with plane waves for the representation of the valence states. Local functions of s, p, and d symmetry at the Ta sites and of s and p symmetry on the Se sites, respectively, were combined with plane waves up to 26 Ry. The exchange-correlation functional was treated in the local-density approximation (LDA), and spin-orbit interaction was taken into account consistently in the calculation of both electronic and phonon properties.

To resolve fine features in our calculations related to the Fermi surface geometry, Brillouin-zone (BZ) integrations were performed with a dense hexagonal 24x24x4 **k**-point mesh (122 points in the irreducible BZ). The standard smearing technique was employed with a Gaussian broadening of 0.1 eV. Tests with the denser **k**-point mesh confirmed sufficient convergence for both phonon energies and line widths. All results were obtained for the experimental hexagonal structure at ambient pressure (a = b = 3.436 Å, c = 12.70 Å)[13] and at a high pressure of p = 23.1 GPa (a = b = 3.2433(4) Å, c = 11.10(3) Å)[14]. The internal parameter was relaxed to z = 0.1207 for ambient and z = 0.1009 for high pressure.

Phonon dispersion on the whole BZ was obtained from DFPT calculations of dynamical matrices of a 12x12x2 **q**-point mesh using standard Fourier interpolation. Furthermore, DFPT gives direct access to electron-phonon coupling (EPC) matrix elements on the same ***k*** and ***q*** meshes, which were used to calculate the isotropic Eliashberg function $\alpha^2F(\omega)$ and the electron-phonon coupling constant $\lambda_{EPC} = 2\int \alpha^2\, F(\omega)/\omega d\omega$. Results for the high-pressure case are shown in Figure 6(d) and correspond to a total $\lambda_{EPC} = 1.03$.

To estimate T_{sc} at high pressures, we solved the linearized gap equation of the Eliashberg theory on the imaginary axis[15]. This equation takes as input the Eliashberg function $\alpha^2F(\omega)$ and the parameter μ^*, which represents an effective Coulomb repulsion. Using a typical value of $\mu^* = 0.1$, we got $T_{sc} = 10.6$ K. When the low-frequency part of $\alpha^2F(\omega)$ below 15 meV is set to zero, λ_{EPC} drops to 0.28, and superconductivity is completely suppressed for $\mu^* = 0.1$. Even for $\mu^* = 0$, T_{sc} would be only 2.5 K. This indicates that the low-frequency modes including the soft branch contribute significantly to the pairing strength.

We assessed the origin of the structural instability for ambient pressure lattice constants by calculating the electronic contribution to the linewidths of phonon modes,

$$\gamma_{EPC}^{\boldsymbol{q}} = \pi\omega_{q\lambda}\sum_{\vec{k}}\left|g_{\vec{k}+\vec{q},\vec{k}}^{q\lambda}\right|^2 \delta(\epsilon_{\vec{k}}-\epsilon_F)\delta(\epsilon_{\vec{k}+\vec{q}}-\epsilon_F) \quad (1),$$

where $g_{\vec{k}+\vec{q},\vec{k}}^{\vec{q}\lambda}$ is the EPC matrix elements for a phonon mode λ at a wave vector $\vec{q}$, $\omega_{q\lambda}$ is the mode's energy and $\sum_{\vec{k}}\delta(\epsilon_{\vec{k}}-\epsilon_F)\delta(\epsilon_{\vec{k}+\vec{q}}-\epsilon_F)$ gives the number of electronic states at the Fermi level connected by the phonon wave vector $\vec{q}$, i.e., the electronic joint density-of-states (eJDOS), which is practically equivalent to the nesting function[16] and the imaginary part of the static electronic susceptibility in the constant matrix-element approximation[17]. Generally, the wave vector of the phonon softening is connected to a maximum in the real part of the susceptibility[18] which has been confirmed for 2*H*-$TaSe_2$.[17] However, the presence of a strongly momentum-dependent line width of the soft mode [see Fig. 6(b)] indicates that, for 2*H*-$TaSe_2$, the location of the phonon softening is also linked to a Fermi surface nesting geometry and, thus, can be investigated via the eJDOS.

Our calculations including spin-orbit coupling predict four bands to make up the Fermi surface of 2*H*-$TaSe_2$, where always two bands are degenerate. Thus, we have essentially two bands crossing the Fermi level [Fig. S4(a)] in agreement with previous calculations[17]. The calculated total eJDOS [Fig. S4(b)] agrees well with the imaginary

part of the electronic susceptibility shown in Figure 4(a) of reference [17]. In particular, our calculations also predict the strongest nesting feature at $\boldsymbol{q} = (1/3,1/3,0)$. However, we showed in previous work that it can be instructive to look into the EPC properties related to electronic transitions between different bands of the Fermi surface[19]. For this purpose we show the partial eJDOSs for all possible band transitions of the two bands making up the Fermi surface in Figures S4(c)-(e). We see that the nesting at $\boldsymbol{q} = (1/3,1/3,0)$ is mostly due to intra-band scattering of band #2 [Fig. S4(e)], whereas the partial eJDOS based on inter-band scattering between bands 1 and 2 shows a maximum close to $\boldsymbol{q}_{CDW}$ [Fig. S4(d)].

EPC in 2*H*-$TaSe_2$ is not restricted only to the CDW soft mode. Still, $\gamma_{EPC}^{\boldsymbol{q}}$ summed over all phonon modes shows a clear peak near $\boldsymbol{q}_{CDW}$ along $\Gamma - M$ but features also an increase towards the zone center [solid line in Fig. S5(a)]. Our calculations allow to distinguish contributions to the sum over all phonons of $\gamma_{EPC}^{\boldsymbol{q}}$ related to electronic transitions between different bands. The results [dashed lines in Fig. S5(a)] show that the contribution related to interband scattering $1 \rightleftharpoons 2$ is responsible for the peak near $\boldsymbol{q}_{CDW}$ whereas contributions related to intraband scattering of bands 1 and 2, i.e., $1 \rightleftharpoons 1$ and $2 \rightleftharpoons 2$, explain the increased EPC approaching the zone center. The comparison with the calculated linewidth due to EPC of the soft mode [Fig. S5(b)] suggests that the soft mode couples primarily to electronic interband transitions of bands $1 \rightleftharpoons 2$.

Hence, we have to consider mainly the eJDOS of the interband transitions $1 \rightleftharpoons 2$ [Fig. S4(d)] if we want to understand the EPC properties of the CDW soft phonon mode. Figure S6 shows a comparison of the momentum dependences of $\gamma_{EPC}^{\boldsymbol{q}}$ of the soft mode (solid line) and the partial eJDOS related to interband scattering $1 \rightleftharpoons 2$ (dashed line) along the $\Gamma - M$ line. For a $\boldsymbol{q}$-independent EPC matrix element g we would expect that $\gamma_{EPC}^{\boldsymbol{q}}$ and the relevant eJDOS have similar momentum dependences according to equation (1). In Figure S6, we find this scenario around $\boldsymbol{q}_{CDW}$ but a peak in the partial eJDOS closer to the zone center is not reflected in $\gamma_{EPC}^{\boldsymbol{q}}$ of the soft mode.

We conclude that the periodicity of the CDW order in 2*H*-$TaSe_2$ is governed by an interplay of EPC matrix elements and Fermi surface topology. First, the matrix elements are such that the soft phonon mode only couples to electronic scattering events between bands 1 and 2 [see Fig. S5 and discussion above]. The eJDOS of this particular scattering channel shows a peak near $\boldsymbol{q}_{CDW}$ and suggests that the Fermi surface topology plays an important role in defining the CDW periodicity, i.e., $\boldsymbol{q}_{CDW}$. However, a certain momentum dependence of the EPC matrix element even for this particular scattering channel is visible.

References

1 Li, L., Deng, X., Wang, Z., Liu, Y., Abeykoon, M., Dooryhee, E., Tomic, A., Huang, Y., Warren, J. B., Bozin, E. S., Billinge, S. J. L., Sun, Y., Zhu, Y., Kotliar, G. & Petrovic, C. Superconducting order from disorder in 2H-$TaSe_{2-x}S_x$. *npj Quantum Materials* **2**, 11, doi:10.1038/s41535-017-0016-9 (2017).

2 Xu, S., Liu, Z., Yang, P., Chen, K., Sun, J., Dai, J., Yin, Y., Hong, F., Yu, X., Xue, M., Gouchi, J., Uwatoko, Y., Wang, B. & Cheng, J. Superconducting phase diagrams of S-doped 2*H*-$TaSe_2$ under hydrostatic pressure. *Physical Review B* **102**, 184511, doi:10.1103/PhysRevB.102.184511 (2020).

3 Said, A. H., Sinn, H., Toellner, T. S., Alp, E. E., Gog, T., Leu, B. M., Bean, S. & Alatas, A. High-energy-resolution inelastic X-ray scattering spectrometer at beamline 30-ID of the Advanced Photon Source. *Journal of Synchrotron Radiation* **27**, 827-835 (2020).

4 Toellner, T. S., Alatas, A. & Said, A. H. Six-reflection meV-monochromator for synchrotron radiation. *Journal of Synchrotron Radiation* **18**, 605-611 (2011).

5 Said, A. H., Sinn, H. & Divan, R. New developments in fabrication of high-energy-resolution analyzers for inelastic X-ray spectroscopy. *Journal of Synchrotron Radiation* **18**, 492-496 (2011).

6 Fåk, B. & Dorner, B. Phonon line shapes and excitation energies. *Physica B: Condensed Matter* **234-236**, 1107-1108 (1997).

7 Shirane, G., Shapiro, S. & Tranquada, J. *Neutron Scattering with a Triple-Axis Spectrometer*. (2002).

8 Hill, H. M., Chowdhury, S., Simpson, J. R., Rigosi, A. F., Newell, D. B., Berger, H., Tavazza, F. & Hight Walker, A. R. Phonon origin and lattice evolution in charge density wave states. *Physical Review B* **99**, 174110 (2019).

9 Rossnagel, K., Rotenberg, E., Koh, H., Smith, N. V. & Kipp, L. Fermi surface, charge-density-wave gap, and kinks in *2H*-$TaSe_2$. *Physical Review B* **72**, 121103 (2005).

10 Borisenko, S., Kordyuk, A., Yaresko, A., Zabolotnyy, V., Inosov, D., Schuster, R., Büchner, B., Weber, R., Follath, R., Patthey, L. & Berger, H. Pseudogap and Charge Density Waves in Two Dimensions. *Physical Review Letters* **100**, 196402 (2008).

11 Inosov, D. S., Zabolotnyy, V. B., Evtushinsky, D., Kordyuk, A., Buechner, B., Follath, R., Berger, H. & Borisenko, S. Fermi surface nesting in several transition metal dichalcogenides. *New J Phys* **10**, 125027 (2008).

12 Inosov, D., Evtushinsky, D., Zabolotnyy, V., Kordyuk, A., Büchner, B., Follath, R., Berger, H. & Borisenko, S. Temperature-dependent Fermi surface of 2H-$TaSe_2$ driven by competing density wave order fluctuations. *Physical Review B* **79**, 125112 (2009).

13 Moncton, D. E., Axe, J. D. & DiSalvo, F. J. Neutron scattering study of the charge-density wave transitions in 2H-$TaSe_2$ and 2H-$NbSe_2$. *Physical Review B* **16**, 801-819 (1977).

14 Shen, X., Tymoshenko, Y. V., Haghighirad, A. A., Lacmann, T., Garbarino, G. & Weber, F. Suppression of charge-density wave order in *2H*-$TaSe_2$ by pressure. *experiment at ESRF*, doi:10.15151/ESRF-ES-902987807 (2022).

15 Bergmann, G. & Rainer, D. The sensitivity of the transition temperature to changes in $\alpha^2F(\omega)$. *Zeitschrift für Physik* **263**, 59-68 (1973).

16 Kasinathan, D., Kuneš, J., Lazicki, A., Rosner, H., Yoo, C. S., Scalettar, R. T. & Pickett, W. E. Superconductivity and Lattice Instability in Compressed Lithium from Fermi Surface Hot Spots. *Physical Review Letters* **96**, 047004 (2006).

17 Johannes, M. D. & Mazin, I. I. Fermi surface nesting and the origin of charge density waves in metals. *Physical Review B* **77**, 165135 (2008).

18 Chan, S. K. & Heine, V. Spin density wave and soft phonon mode from nesting Fermi surfaces. *Journal of Physics F: Metal Physics* **3**, 795 (1973).

19 Weber, F., Rosenkranz, S., Castellan, J. P., Osborn, R., Karapetrov, G., Hott, R., Heid, R., Bohnen, K. P. & Alatas, A. Electron-Phonon Coupling and the Soft Phonon Mode in $TiSe_2$. *Physical Review Letters* **107**, 266401 (2011).
